# Diet-induced obesity impairs hypothalamic glucose sensing but not glucose hypothalamic extracellular levels, as measured by microdialysis

**DOI:** 10.1038/nutd.2015.12

**Published:** 2015-06-15

**Authors:** I S de Andrade, J C S Zemdegs, A P de Souza, R L H Watanabe, M M Telles, C M O Nascimento, L M Oyama, E B Ribeiro

**Affiliations:** 1 Departamento de Fisiologia, Disciplina de Fisiologia da Nutrição, Universidade Federal de São Paulo (UNIFESP), São Paulo, Brazil; 2 Departamento de Ciências Biológicas, Universidade Federal de São Paulo (UNIFESP), Diadema, Brazil

## Abstract

**Background/Objectives:**

Glucose from the diet may signal metabolic status to hypothalamic sites controlling energy homeostasis. Disruption of this mechanism may contribute to obesity but its relevance has not been established. The present experiments aimed at evaluating whether obesity induced by chronic high-fat intake affects the ability of hypothalamic glucose to control feeding. We hypothesized that glucose transport to the hypothalamus as well as glucose sensing and signaling could be impaired by high-fat feeding.

**Subjects/methods:**

Female Wistar rats were studied after 8 weeks on either control or high-lard diet. Daily food intake was measured after intracerebroventricular (i.c.v.) glucose. Glycemia and glucose content of medial hypothalamus microdialysates were measured in response to interperitoneal (i.p.) glucose or meal intake after an overnight fast. The effect of refeeding on whole hypothalamus levels of glucose transporter proteins (GLUT) 1, 2 and 4, AMPK and phosphorylated AMPK levels was determined by immunoblotting.

**Results:**

High-fat rats had higher body weight and fat content and serum leptin than control rats, but normal insulin levels and glucose tolerance. I.c.v. glucose inhibited food intake in control but failed to do so in high-fat rats. Either i.p. glucose or refeeding significantly increased glucose hypothalamic microdialysate levels in the control rats. These levels showed exacerbated increases in the high-fat rats. GLUT1 and 4 levels were not affected by refeeding. GLUT2 levels decreased and phosphor-AMPK levels increased in the high-fat rats but not in the controls.

**Conclusions:**

The findings suggest that, in the high-fat rats, a defective glucose sensing by decreased GLUT2 levels contributed to an inappropriate activation of AMPK after refeeding, despite increased extracellular glucose levels. These derangements were probably involved in the abolition of hypophagia in response to i.c.v. glucose. It is proposed that ‘glucose resistance' in central sites of feeding control may be relevant in the disturbances of energy homeostasis induced by high-fat feeding.

## Introduction

Obesity is a multifactorial condition that has grown worldwide as a result of increased intake of palatable high-caloric foods allied to reduced physical activity.^1, 2^ Diets rich in either polyunsaturated or saturated fats reportedly associated with increased adiposity in rodents.^3, 4, 5, 6^ Understanding to which extent diet is a relevant factor in the etiology of obesity is essential for its prevention and treatment.

Central nervous system mechanisms have a pivotal role in the regulation of energy intake and expenditure.^7, 8, 9^ In obesity, several brain energy homeostasis mechanisms are reportedly altered.^6, 10, 11, 12^

Nutrients may act at the hypothalamus to signal metabolic status and thus influence food intake as well as glucose and energy homeostasis.^13, 14, 15, 16^ The medial hypothalamic region, which includes the ventromedial (VMH) and the arcuate (ARC) nuclei, is a key site for regulation of food intake and glycemia and has been shown to have glucosensing neurons able to alter their firing rate in response to variations in cerebral glucose levels. Both glucose-excited and glucose-inhibited neurons, as they respond to glucose increments with increased or decreased activity, respectively, have been reported in the medial hypothalamus.^17, 18^ Glucose-inhibited neurons reportedly express the orexigenic mediator neuropeptide Y (NPY) while glucose-excited neurons have been shown to produce the anorexigenic mediator proopiomelanocortin (POMC).^13, 15^ Intracerebroventricular (i.c.v.) or intra-ARC glucose injections reportedly inhibited feeding while decreasing NPY and agouti-related protein expression.^19, 20, 21^ Failure of these glucose-induced responses could have a role in the pathogenesis of obesity.^22^

Glucose uptake across the blood–brain barrier and into brain neurons occurs via glucose transporter proteins (GLUT) 1, 2, 3 and 4. Expressed in micro vessels, tanycytes, astrocytes and neurons, these transporters have been shown to have relevant roles not only in glucose uptake but also in glucose sensing. A special part has been attributed to hypothalamic GLUT2 in the glucose sensing involved in feeding regulation mechanisms operating at the hypothalamus.^16, 23, 24, 25^ Moreover, the AMP-activated protein kinase (AMPK) has been suggested as a mediator of the hypothalamic glucose actions leading to feeding regulation. AMPK is activated by phosphorylation in response to increased AMP: ATP ratio and it acts to stimulate energy-yielding processes, such as food intake.^16, 26, 27^

The present experiments were aimed at evaluating whether chronic high-fat intake affects the ability of glucose to control feeding by acting at the hypothalamus. We hypothesized that glucose transport to the hypothalamus as well as glucose sensing and signaling could be impaired by high-fat feeding. We thus performed brain microdialysis and immunoblotting experiments to measure the effect of a meal intake on hypothalamic extracellular glucose levels and GLUTs and AMPK levels. A preliminary form of this work has been previously presented.^28^

## Materials and methods

### Animals and diets

All procedures were approved by the Committee on Animal Research Ethics of the Federal University of São Paulo. Ninety Wistar female rats were kept six per cage under controlled temperature (24±1 °C) and lights on from 0600 to 1800 hours, with free access to water. They were randomly assigned to receive either standard rat chow (control diet: 4.5% lipid, 22.7% protein, 35.9% carbohydrate (w/w), 2.7 Kcal g^−1^, Nuvilab, Brazil) or lard-enriched chow (high-fat diet: 23.5% lipid, 27.6% protein, 21.2% carbohydrate (w/w), 4.1 Kcal g^−1^), from 2 to 4 months of age. The high-fat diet was prepared by adding, to the control diet (w/w), 20% lard, 10% sucrose and 20% casein (to obtain a protein/energy ratio similar to that of the control diet). Body weight and daily food and energy intake were monitored weekly.

All experiments were reproduced at least one time. Sample sizes were kept to a minimum whenever possible

### Effect of high-fat intake for 8 weeks on body fat and serum parameters

The animals were fasted for 6 h and killed around noon. Serum glucose was determined in 10 μl aliquots by enzymatic method with detection limit of 0.41 mg dl^−1^ (Glucose Pap Liquiform, Labtest Diagnostica, São Paulo, Brazil). Insulin levels were determined by radioimmunoassay with detection limit of 0.1 ng ml^−1^(Rat Insulin, RIA kit, Millipore, Bedford, MA, USA). Leptin levels were determined by enzyme-linked immunosorbent assay (Rat Leptin, Millipore) with detection limit of 0.04 ng ml^−1^.

Carcass lipids were extracted^29^ and determined gravimetrically.^30^

### Effect of i.c.v. glucose on food intake

Rats were anesthetized with ketamine/xylazine (66.6/13.3 mg kg^−1^, i.p.) and stereotaxically implanted with a guide cannula aimed at the right lateral ventricle (A −1.2 mm, L −1.6 mm, 2.5 mm ventral to bregma).^31^ At the end of surgery, they received subcutaneous injection of antibiotic (benzetacil 1.200.000UI, Teuto/Pfizer, Anápolis, Goias, Brazil) and oral analgesic (Ibuprofen, 25 mg kg^−1^) and kept in individual cages for recovery for 7 days, with free access to water and diet.

On the day of the experiment, the animals were weighed and the food was withdrawn at noon. At 1800 hours they were i.c.v. injected with 5 μl of either vehicle (artificial cerebrospinal fluid) or glucose solution (50 or 100 μg). Pre-weighed food cups were introduced and food consumption was recorded after 2, 12 and 24 h. Each animal was injected twice, receiving vehicle or glucose, on separate days, 2 days apart. They were randomly divided so that half the animals received vehicle as the first injection and the other half received glucose as the first injection. Cannula placement was confirmed by the correct distribution of 5 μl of Evans blue dye administered after deep anesthesia.

### Effect of i.p. glucose or refeeding on glucose levels in VMH microdialysates

The rats were stereotaxically implanted with a guide cannula aimed at the right hypothalamic VMH nucleus (AP −2.5 mm, L −0.6 mm, DV −7.9 mm ventral to bregma)^31^ and received antibiotic and analgesic.

Seven days later, around 1800 hours, the rats were weighed, food was removed and the microdialysis probe was inserted into the guide cannula. The probes were custom constructed as previously described,^32, 33^ with 2.0 mm of effective membrane length and 13000 Da cut-off. *In vitro* relative probe recovery for glucose was 6.5±0.95%.

The probe inlet was connected to a micro-infusion pump (CMA-Harvad Apparatus, Kista, Stockholm, Sweden) and the animal was connected to a swivel system, allowing constant probe perfusion (1.5 μl min^−1^) with cerebrospinal fluid (145 mm NaCl, 2.7 mM KCl, 1.0 mM MgCl2, CaCl2 1.2 mM, 2.0 mM Na2HPO4, pH 7.4).^34^ After 16 h of fasting, 30-min microdialysate samples were collected and stored at −20 °C until analysis of glucose content, performed no later than the next day. After the collection of three baseline samples, the control and the high-fat groups received a known amount of the control or the high-fat diet, respectively. For this, the food pellets were grounded and mixed with water (1:1). Food was available for 30 min and the amount eaten was recorded. Twelve additional dialysate samples were collected following the withdrawal of the food.

Other rats submitted to the same microdialysis procedures received, instead of food, an i.p. injection of glucose (2 g kg^−1^).

Glucose was directly assayed in 40 μl microdialysate samples by the enzymatic method described above.

### Effect of i.p. glucose or refeeding on serum glucose levels

Serum glucose was determined in parallel experiments in rats implanted with a venous catheter at 1700 hours. After 16 h of fasting, they were weighed and one blood sample (0.1 ml) was collected. The same procedures used in the microdialysis experiments were then performed. Either a known amount of food mash was made available for 30 min or 2 g kg^−1^ glucose was administered i.p. Blood samples were collected every 30 min and serum glucose analyzed as above.

### Effect of refeeding on hypothalamic protein expression

Additional rats were killed either in the fasted state or 90 min after refeeding. The hypothalami were homogenized and 50 μg of protein were resolved in SDS–polyacrylamide gel electrophoresis, transferred to nitrocellulose membranes and incubated with primary antibody against GLUT1 (sc 1603), GLUT4 (sc 1606), AMPKα1/2 (sc 25792), (Santa Cruz Biotechnology, Dallas, TX, USA), GLUT2 (ab9256, Abcam, Cambridge, UK) or pAMPK^Thr172^ (#2531, Cell Signaling Technology, Inc., Danvers, MA, USA). The blots were incubated with peroxidase-conjugated secondary antibodies and specific bands were detected by chemiluminescence (ECL reagent, GE Healthcare Bio-sciences, Pittsburgh, PA, USA). For evaluation of protein loading, all membranes were stripped and re-blotted with anti-alpha-tubulin primary antibody. Band intensities were quantified by optical densitometry (Scion Image software, Scion Corporation, Frederick, MD, USA). The results are expressed in arbitrary densitometry units.

### Histology

For verification of positioning of microdialysis membranes, at the termination of the experiments all animals were deeply anesthetized and perfused with 0.9% phosphate-buffered saline followed by 4% paraformaldehyde. Brains were removed and 45 μm sections were stained with cresyl violet. Only data from rats with correct membrane placement were included in the analysis.

### Data analysis

The results are expressed as mean±s.e.m. The basal glucose levels are expressed as their absolute dialysate content and were not corrected by *in vitro* probe recovery. Due to inter-individual variations, a mean baseline level (100% value) was calculated by averaging glucose content of the three pre-stimuli microdialysate samples. All post-stimuli glucose values are expressed as percentage of baseline. The area under the curve relating glucose levels to time was calculated by the trapezoidal rule (GraphPad Software, Inc., San Diego, CA, USA) and compared by Student's *t*-test.

Data of microdialysate and serum glucose levels, food intake after i.c.v. glucose, and body weight and food intake throughout diet treatment were analyzed by two-way analysis of variance followed by Bonferroni test. All other comparisons between the two diets were performed by Student's *t-*test. Hypothalamic protein expression was analyzed by one-way analysis of variance followed by Bonferroni test. Statistical significance was set at *P*<0.05 (two sided).

## Results

### Effect of high-fat intake for 8 weeks on food intake, body fat and serum parameters

The high-fat group had decreased food intake with normal energy intake throughout treatment. Cumulative intake of lipids was higher in the high-fat rats (10.73±0.52%) than in controls (2.69±0.04%) while that of carbohydrates was lower (9.68±0.46%) than in controls (21.48±0.32%). Body weight was increased in high-fat rats since week 5. At week 8, the high-fat group had increased carcass fat (16.6±0.9 vs 10.2±0.4 g per 100 g b.w.), serum leptin (3.0±0.6 vs 0.9±0.2 ng dl^−1^) and cumulative body weight gain (75.9±2.5 vs 63.5±1.8 g), with normal serum glucose and insulin levels (data not shown).

### Effect of i.c.v. glucose injection on food intake

**Figure 1:**
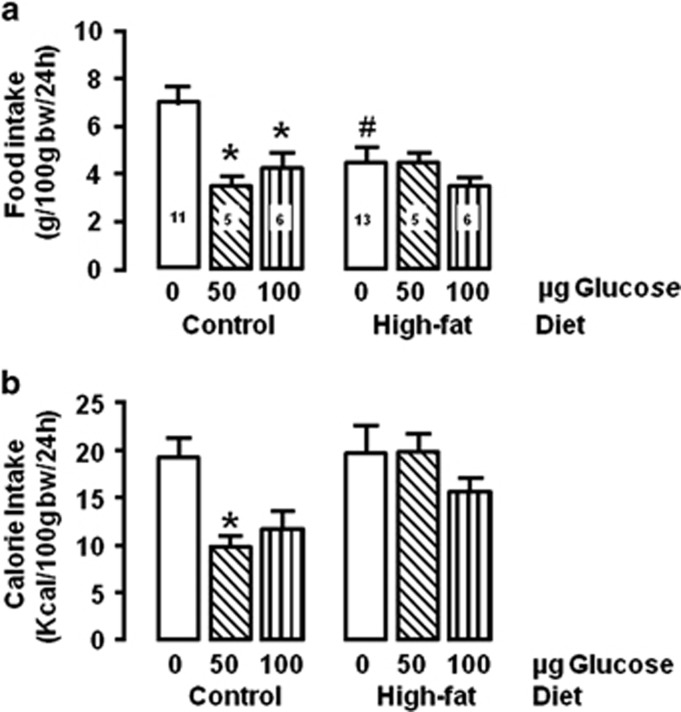
Food mass and calorie intakes after 24 h of intracerebroventricular glucose or vehicle injection in control or high-fat groups. *P<0.05 vs respective vehicle; ^#^*P*<0.05 vs control group. The numbers inside the bars indicate the number of animals.

I.c.v. glucose inhibited 24 h food mass intake (*F*_(2,38)_=6.49, *P*=0.0038), with a significant interaction with diet *(F*_(2,38)_=4.41, *P*=0.0189). In the control group, both glucose doses significantly inhibited intake (50 μg *P*<0.001; 100 μg *P*<0.01). Contrarily, no significant food inhibition after glucose occurred in the high-fat group (Figure 1a).

Caloric intake was affected by glucose (*F*_(2,38)_=3.69, *P*=0.0337). In the control group, the low dose caused a significant feeding inhibition (*P*<0.05), while no significant feeding inhibition occurred in the high-fat group (Figure 1b).

### Effect of i.p. glucose on glucose levels in serum and in VMH microdialysates

**Figure 2:**
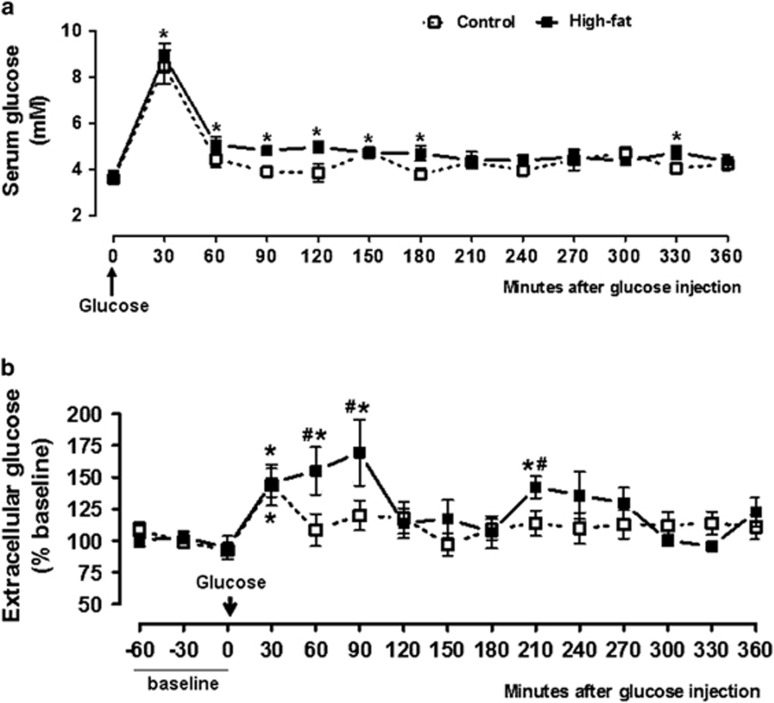
Serum glucose (**a**) and VMH microdialysate glucose (**b**), before and after i.p. glucose injection. (**a**) Control: *n*=6 animals, high-fat: *n*=6 animals. *P<0.05 vs 0 min, ^#^*P*<0.05 vs control group. (**b**) Control: *n*=9 animals; high-fat: *n*=7 animals. *P<0.05 vs baseline; ^#^*P*<0.05 vs control group.

Figure 2a shows significant effects of diet (*F*_(1,117)_=9.207, *P*=0.003) and time (*F*_(12,117)_=29.62, *P*<0.0001) on serum glucose. Thirty minutes after i.p. glucose, serum glucose increment increased similarly in both groups (*P*<0.0001). Levels returned to normal in the control group at 60 min, while the high-fat group showed increased levels until 180 min and at 330 min. The area under the curve relating glucose levels to time was similar between control (418±61) and high-fat group (525±92) for the 360 min after glucose administration (*P*=0.18).

Baseline microdialysate absolute content was similar between the groups (control: 120±18; high-fat: 130±19 ng per 10 μl). After i.p. glucose, microdialysate levels showed a significant effect of diet (*F*_(1,182)_=8.894, *P*=0.0033) and time (*F*_(14,182)_=3.151, *P*=0.0002). Figure 2b shows that, in the control group, VMH glucose increased significantly at 30 min (43±16%, *P*<0.01). The high-fat group had significantly increased VMH glucose at 30 min (46±11%), 60 min (55±18%), 90 min (69±26%) and at 210 min (42±8%) post injection.

The high-fat group presented enhanced levels than those of the control group throughout the experiment, although with statistical significance only at the times 60, 90 and 210 min. The area under the curve relating glucose levels to time was significantly higher in the high-fat group (12750±1898) than in the control group (6765±1991) (*P*=0.0281).

### Effect of refeeding on glucose levels in serum and in VMH microdialysates

The 30-min food intake was lower in the high-fat group (2.16±0.32 g) as compared with the control group (3.24±0.46 g, *P*=0.048). The control group had higher intakes of carbohydrates (1.16±0.17% vs 0.42±0.07%, *P*<0.0001) and fiber (0.61±0.09% vs 0.15±0.02%, *P*<0.0001) and a lower intake of fat (0.15±0.02% vs 0.51±0.08%, *P*<0.001) than those of the high-fat group.

**Figure 3:**
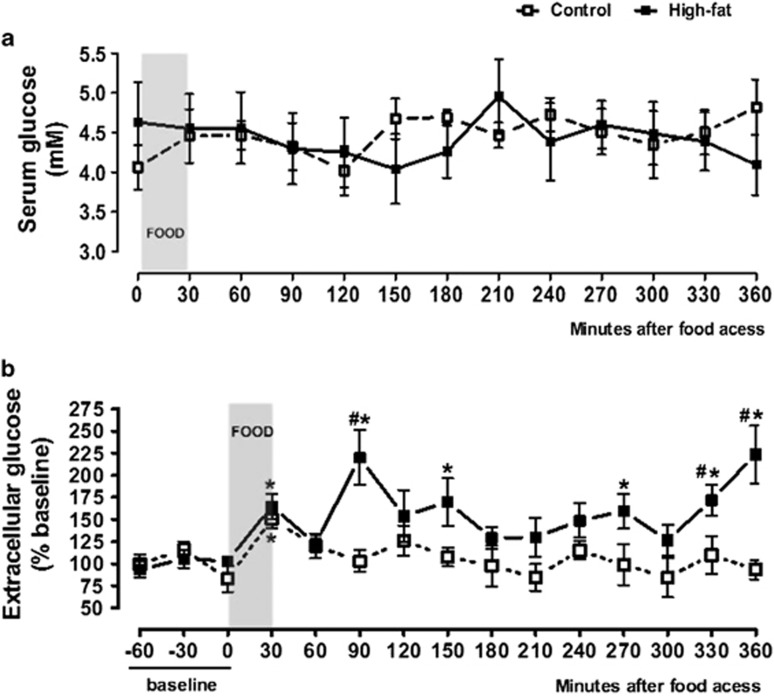
Serum glucose (**a**) and VMH microdialysate glucose (**b**), before and after food intake. (**a**) Control: *n*=7 animals; high-fat: *n*=8 animals. (**b**) Control: *n*=10 animals; high-fat: *n*=11 animals. *P<0.05 vs baseline; ^#^*P*<0.05 vs control group.

Figure 3a shows the effect of food intake on serum glucose. There were no significant effects of diet (*F*_(1,174)_=0.1016, *P*=0.7503), time (*F*_(12,174)_=0.3220, *P*=0.9846) or diet–time interaction (*F*_(12,174)_=0.6161, *P*=0.8267).

VMH extracellular glucose levels were affected by diet (*F*_(1,233)_=40.23, *P*<0.0001), time (*F*_(14,233)_=3.082, *P*=0.0002) and diet–time interactions (*F*_(14,233)_=2.263, *P*=0.0066). Figure 3b shows that microdialysate glucose levels were increased after feeding in both the control and the high-fat groups. In controls, the levels were significantly increased only at 30 min (50±10% from baseline, *P*<0.05). The increases were more prominent after the intake of the high-fat diet with higher levels at 30 min (65±14%, *P*<0.05), 90 min (120±31%, *P*=0.001), 150 min (69±26%, *P*=0.01), 270 min (59±19%, *P*=0.05), 330 min (72±18%, *P*=0.01) and 360 min (123±32%, *P*=0.001) after refeeding. The levels differed significantly between high-fat and control rats at 90 min (*P*=0.001), 330 min (*P*=0.01) and 360 min (*P*=0.001). The area under the curve relating glucose levels to time were higher in the high-fat (18700±3763) than in the control group (10630±1080) (*P*=0.0271).

### Effect of refeeding on hypothalamic protein expression

**Figure 4:**
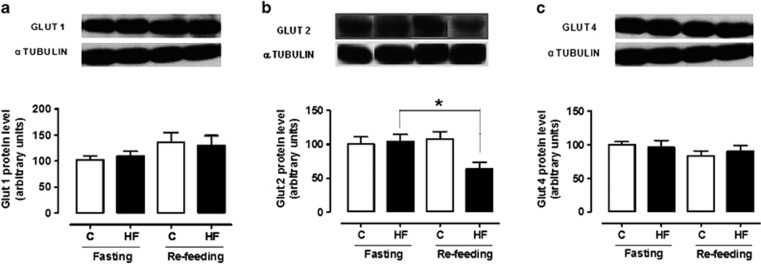
Hypothalamic protein levels of Glut1 (**a**) and Glut2 (**b**) and Glut 4 (**c**) in control and high-fat groups either in the fasted state or after refeeding. *n*=5–7 animals. *P<0.05 vs the respective fasted group.

**Figure 5:**
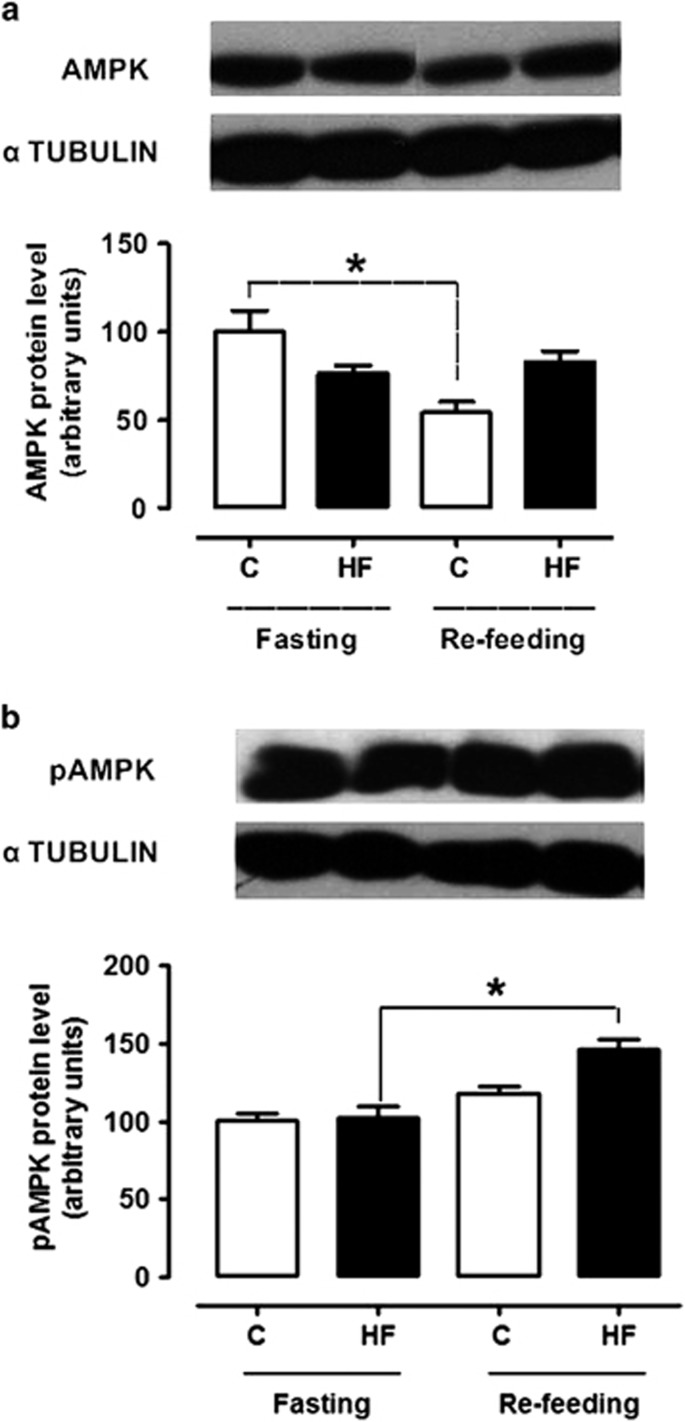
Hypothalamic protein levels of AMPK (**a**) and pAMPK (**b**) in control and high-fat groups either in the fasted state or after refeeding. *n*=4–5 animals *P<0.05 vs the respective fasted group.

Figures 4 and 5 show that, in the fasted state, the hypothalamic levels of Glut1, Glut2, Glut4, AMPK and pAMPK were similar between the control and the high-fat groups. Refeeding failed to affect Glut1 and Glut4 expression in both groups but it decreased Glut2 expression in the high-fat group (*F*_(3,25)_=3.799, *P*=0.025). Refeeding decreased AMPK levels in the control group (*F*_(3,18)_=6.69, *P*=0.004) while it increased pAMPK levels in the high-fat group (*F*_(3,17)_=14.47, *P*=0.0001).

## Discussion

These experiments confirmed our preliminary data and other authors' reports showing feeding inhibition by central glucose administration to normal animals.^19, 20, 21, 35, 36^ Among the multiple central nervous system pathways controlling feeding, a direct effect of glucose is likely to be of physiological importance but this has not been well established. Moreover, while the importance of the medial hypothalamus in feeding control is well recognized,^9, 11, 37^ the role played by glucose signaling during diet-induced obesity has not been ascertained. The present demonstration that the hypophagic action of central glucose was abolished in rats made obese by high-fat intake, indicates that defective central glucose action may be relevant in this obesity model.

This notion is in accordance with earlier findings that, unlike dietary obesity-resistant rats, in obesity-prone rats glucose injected into the carotid artery failed to induce hypothalamic neuronal activation.^38^ Functional magnetic resonance imaging studies have shown alteration of the medial hypothalamic response to oral glucose in obesity, although with contradictory results. While the functional magnetic resonance imaging signal has been found to decrease in lean humans and rats following glucose ingestion, obese men showed attenuation while obese rats showed accentuation of the signal decrement.^39, 40^ In Zucker obese rats, electric activity in the ARC nucleus has been shown to be overstimulated by intracarotid glucose and this hypersensitivity was associated with exacerbated insulin secretion, even in the absence of glycemic changes.^41^ These data corroborate the present observations of disrupted glucose action in the hypothalamus of obese rats, although there is no uniformity among these studies concerning the alteration present in obese individuals.

It is accepted that the hypothalamus responds to glucose through glucose-sensing neurons. In obese rats, both glucose-excited and glucose-inhibited neurons have been found to be less numerous and to abnormally respond to glucose variations.^17, 38^

Around 40% of ARC nucleus glucose-inhibited neurons reportedly express the orexigenic peptide NPY^42^ and expression of the anorexigenic precursor POMC by glucose-excited neurons has been demonstrated.^43^ It is thus reasonable to suggest that the physiological mechanisms of the hypophagic response to i.c.v. glucose, as performed herein, would include inhibition by glucose of NPY-expressing neurons and stimulation of POMC-expressing neurons. The failure of the high-fat rats to show intake inhibition after i.c.v. glucose indicates that these responses were defective, a suggestion compatible with the knowledge of exacerbated activity of NPY neurons and decreased activity of POMC neurons in obesity.^9, 44^ The present finding of hyperleptinemia in the high-fat rats, indicative of leptin resistance, agrees with this hypothesis.

Because we administered glucose directly in the cerebral ventricle, the doubt remained of whether the ability of glucose to reach the brain would be modified in the obese animals after a meal. This is a relevant aspect that could influence glucose ability to control food intake by a central effect. We then performed microdialysis experiments to evaluate whether the high-fat diet affected the amount of glucose reaching the medial hypothalamus. We first observed that, in the fasted state, serum glucose and baseline extracellular glucose content in VMH microdialysates were not modified by the long-term intake of the high-fat diet. When an i.p. glucose bolus was administered, serum glucose levels rose similarly between the control and the high-fat groups, with a short-lasting 145% glycemic increase, which was evident only in the 30 min post-injection sample. This indicates the absence of overt glucose intolerance in the high-fat rats.

Extracellular glucose content in VMH microdialysates also increased after the i.p. glucose load in both control and high-fat rats. However, unlike the similar serum glycemic effect between the groups, the hypothalamus showed divergent responses. In the control rats, a significant 43% increment in VMH microdialysate glucose occurred only in the 30-min sample and values failed to significantly differ from baseline afterwards. Contrastingly, the high-fat rats had a more accentuated and longer lasting increase in extracellular glucose than the control ones. Previous studies have shown that hyperglycemia induced by intravenous glucose infusion for 90 min was associated with increased levels of extracellular glucose in the brain cortex of normal rats,^45^ as well as in the hippocampus of normal human subjects.^46^

Having found that the high-fat rats did not present impairment of glucose reaching the hypothalamus after i.p. administration, we performed experiments to ascertain the response to the physiological stimulus represented by food intake. Microdialysate glucose levels increased significantly in the control rats, in agreement with other microdialysis studies of the VMH and the lateral hypothalamus of normal rats.^47, 48^ Interestingly, we found that the high-fat rats again responded more pronouncedly than the control ones.

It is important to note that, due to the differences in diet composition and food mass intake during the 30 min of food access, the high-fat group had a lower intake of carbohydrates and fiber and a higher intake of fat than those of the control group. Since fiber slows food absorption and fat decreases food glycemic index^49^ it is possible that the rate of carbohydrate absorption was not affected in the high-fat rats, in accordance with the finding of absence of significant glycemic variations in both groups. These data suggest that neither the amount of glucose ingested nor the feeding-induced glycemic variations were directly related to the variations of VMH glucose levels. Absence of such direct relations has been previously reported for feeding-induced glucose levels in the hypothalamus.^47^

The present observations in the VMH agree with our previous demonstration of exacerbated extracellular glucose increase in the prefrontal cortex of rats fed with high-fat fish oil diet.^50^ An oral glucose overload induced striatal glucose levels to increase more pronouncedly in diabetic than in normal rats.^51^ The present findings are also consistent with the demonstration that hypothalamic glucose concentration increased in diabetic rats.^52^

The above findings thus showed that, after food intake, an excess amount of glucose reached the hypothalamus of the high-fat rats. Since we observed that glucose administered i.c.v. inhibited feeding in the control rats but failed to do so in the high-fat rats, we performed additional experiments aimed at analyzing aspects related to hypothalamic glucose sensing and signaling, by examining the effect of refeeding on GLUT protein and AMPK protein and phosphorylation levels.

We detected no significant feeding-induced variations in GLUT1 and 4 levels in the hypothalamus of control and high-fat rats. Since our animals were fasted for 16 h, these findings agree with earlier work demonstrating absence of variations in hypothalamic glial GLUT1 and GLUT3 protein contents in normal rats fasted for 12 h in comparison to *ad libitum* fed rats, while 24 h of fasting was associated with increased levels of these transporters.^23^ Other authors reported increased GLUT1 and GLUT4 protein levels in the hypothalamus of mice after 48 h of fasting.^53^

On the other hand, the levels of GLUT2 remained unchanged in the control rats while they were decreased by feeding in the high-fat ones. In hypothalamic glial cells of rats, GLUT2 levels reportedly declined after 12 and 24 h of fasting, in relation to *ad libitum* feeding,^23^ indicating a role of this transporter in stimulating food intake during fasting. It is thus apparent that the acute response to refeeding was abnormal in the high-fat group. Absence of GLUT2 has been shown to stimulate daily food intake and to abolish acute feeding responses to glucose and to cytoglucopenia, indicating a role of GLUT2 in hypothalamic glucose sensing.^25^ It is thus likely that our present observation of no feeding inhibition by glucose in the high-fat group was related, at least in part, to an abnormal decline of GLUT2 in the hypothalamus of these obese rats.

The AMPK response to refeeding was also affected in the high-fat group. While the physiological response to refeeding^54^ was present in the control rats, as AMPK levels decreased, the high-fat rats had an opposite response, that is, AMPK levels failed to decrease and pAMPK levels were even increased. It can be suggested that this probably also contributed to the abolition of glucose-evoked hypophagia in the high-fat group. Indeed, AMPK inhibition has been shown to follow central glucose administration and to mediate glucose-induced feeding inhibition.^14, 55, 56^ Absence of AMPK inhibition by leptin has been demonstrated after high-fat feeding.^55, 57^ Since we observed high leptin levels in the high-fat group, and since leptin inhibits VMH glucose-inhibited neurons through AMPK inhibition,^58^ the likely leptin resistance of the high-fat rats may have been of relevance.

Overexpression of GLUT2 by neuroblastoma cells *in vitro* has been shown to accentuate the inhibition of AMPK phosphorylation induced by glucose while AMPK stimulation by cytoglucopenia was attenuated.^26^ These findings indicate that AMPK inhibition by glucose may be dependent on GLUT2 glucose sensing. It is thus possible that the presently observed failure of AMPK to respond properly to glucose in the high-fat group is related to the putative impaired GLUT2 mediation induced by lowered levels of this transporter.

One additional aspect that deserves consideration is the fatty-acid component of the meals used in the present experiments. A decrease in AMPK activity, leading to increased formation of malonyl-CoA through activation of acetyl-CoA carboxylase, has been suggested to mediate feeding inhibition induced by fatty acids at the hypothalamus.^14^ A palatable diet rich in saturated fat prevented the hypophagia induced by the i.c.v. administration of oleic acid.^14^ It is thus possible that the increased level of AMPK activation also affected the mechanism of feeding inhibition induced by fatty acids. Additional experiments are necessary to elucidate this hypothesis.

**Figure 6:**
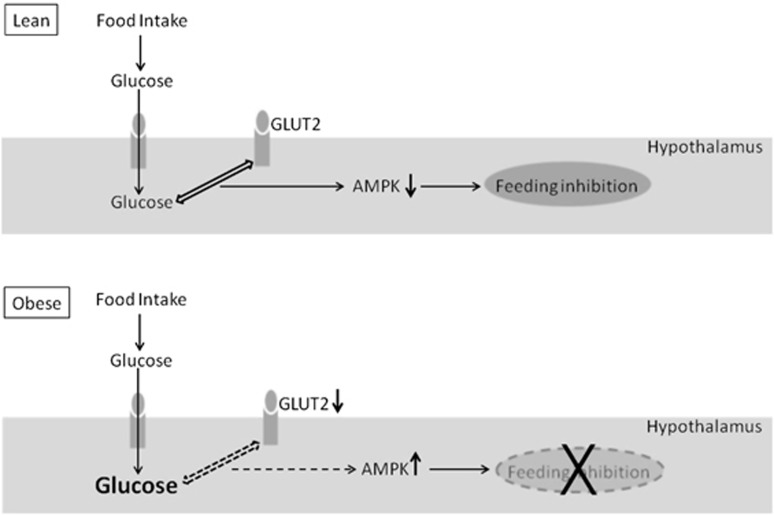
Diagram depicting the responses induced by refeeding in control lean and in dietary obese rats. In lean rats, food-derived glucose reaching the hypothalamus is sensed by GLUT2, leading to decreased AMPK and feeding inhibition. This mechanism is disrupted in the obese rats. Although the feeding-induced hypothalamic glucose levels are in excess, the decreased GLUT2 levels impair glucose sensing, leading to inappropriate AMPK increase and abolition of hypophagia.

In summary, we observed that the feeding inhibition induced by i.c.v. glucose was abolished by long-term high-fat intake. In response to a meal, the high-fat rats showed decreased hypothalamic levels of GLUT2 and increased levels of pAMPK, despite an exacerbated extracellular glucose increase in VMH microdialysates. These findings strongly suggest that a defective glucose sensing by decreased GLUT2 levels contributed to an inappropriate activation of AMPK after refeeding. These derangements were probably involved in the abolition of hypophagia in response to i.c.v. glucose. It is proposed that ‘glucose resistance' in central sites of feeding control may be relevant in the disturbances of energy homeostasis induced by high-fat feeding (Figure 6). Future studies ascertaining, for instance, the effect of high-lard feeding on food intake as well as on the mechanisms of glucose signaling induced by a glucose load in animals overexpressing GLUT2 may contribute to the verification of this hypothesis.
